# Adverse childhood experiences, subsequent negative life events, and their impact on health in occupational rehabilitation patients: a mixed-methods study

**DOI:** 10.3389/fresc.2024.1389337

**Published:** 2024-11-13

**Authors:** Monica Eftedal, Thomas Johansen, Ruby Del Risco Kollerud

**Affiliations:** Norwegian National Advisory Unit on Occupational Rehabilitation, Rauland, Norway

**Keywords:** adverse childhood experiences (ACEs), negative life events, child maltreatment, revictimization, occupational rehabilitation, health, mixed methods

## Abstract

**Introduction:**

Adverse childhood experiences (ACEs) are prevalent globally and can negatively impact an individual's lifespan by not only increasing the likelihood of encountering other negative life events (NLEs), but also escalating the risk of illness, absenteeism due to sickness, unemployment, and reliance on disability benefits in adulthood. Therefore, the objective of this study was to explore the prevalence of ACEs and NLEs, as well as their health impacts among patients undergoing occupational rehabilitation.

**Materials and methods:**

A total of 80 participants diagnosed with musculoskeletal disorders and/or common mental disorders who participated in two occupational rehabilitation programs in Norway were included. Data were collected by questionnaire and in-depth interviews (39 participants) at the start of the intervention. Comparative quantitative and qualitative analysis was conducted between individuals with a history of ACEs and those without these experiences. Thematic analysis was used to identify the impact of ACEs and NLEs on the health of the informants.

**Results:**

Half of the participants reported ACEs. Of these, 18% reported one ACE, 22% reported 2–3 ACEs and 9% reported 4 or more ACEs. Also, 25% were categorized as revictimized. The two groups with ACEs had more NLEs in adulthood compared to those without ACEs (*p* < 0.001), revictimized the most (mean numbers between groups 3.1, 4.5 and 5.9). Furthermore, a history of ACEs was associated with a higher number of reported mental health issues compared to those who had not experienced ACEs (*p* < 0.01). However, there were no significant differences between the two ACE groups. NLEs had a substantial impact on the participants’ current health status, whether they occurred in childhood or adulthood. In adult life, a high workload (psychologically or physically), interpersonal challenges, and financial struggles had an especially negative impact. Additionally, accidents and complications related to surgeries were also significant NLEs causing health problems. For most, there were complex interactions between NLEs and health.

**Conclusions:**

The prevalence of ACEs and NLEs is high among occupational rehabilitation patients. ACEs are associated with subsequent victimization, interpersonal challenges, financial struggles, and increased mental health issues in adulthood. These findings highlight the need for systematic screening and a holistic, individualized approach in occupational rehabilitation programs to potentially mitigate the adverse effects of NLEs on health and work participation.

## Introduction

The term Adverse Childhood Experiences (ACEs) was initially defined by Felitti and colleagues as a combination of child maltreatment encompassing exposure to psychological, physical, and sexual abuse, physical and emotional neglect, and various household dysfunctions during the first 18 years of life (1). The global lifetime prevalence of child maltreatment is widespread, according to the World Health Organization (WHO) (2). The prevalence reported for the WHO regions of Americas and Europe are, respectively (median values), 38% and 14% for psychological abuse, 19% and 10% for physical abuse, 14% and 8% for sexual abuse, and 22% and 10% for neglect. Maltreatment types often overlap, and the cumulative impact often leads to more serious and chronic health outcomes than a single episode of maltreatment (3, 4). Individuals exposed to ACEs are also at an increased risk of encountering other negative life events (NLEs) throughout their lives (4). NLEs in this setting can broadly be defined as any occurrence in a person's life that has changed their life for the worse, either objectively or in their subjective perception (5). However, the prevalence of ACEs and NLEs, as well as their health impacts, remain unexplored among patients undergoing occupational rehabilitation.

Research studies have consistently shown that children who are victims of violence in their family are more likely to experience subsequent victimization in youth and adulthood, including rejection, bullying, physical and emotional violence, especially intimate partner violence (6–8). Moreover, those exposed to any form of ACEs are more prone to experiencing interpersonal problems, work-related issues, absenteeism, unemployment, and financial problems in adulthood (9–13). Also, a multitude of health risk behaviors, such as alcohol consumption, smoking and risk-taking behaviors have been thoroughly documented (13, 14). Regarding health issues and ACEs, ACEs exhibit robust associations with diagnoses pertaining to mental health issues, including depression (1, 15, 16), anxiety disorder (7, 16), posttraumatic stress disorder (16, 17), and suicidal behavior and thoughts (1, 4, 18). A scoping review looking at ACE and associated health outcomes found fewer studies examining physical and general health outcomes than mental health outcomes (19). However, the physical health consequences of ACEs may be as substantial as the mental health consequences (20, 21). For example, strong associations have been found with painful medical conditions (22–24), adult sleep disorders (25), cardiovascular diseases (20), and multimorbidity (26).

The childhood and adult exposures may have both independent, additive, or synergistic effects on adult health and work disability (27). A particular study discovered that synergistic interactions among specific pairs of ACE could account for approximately 30%–40% of the variance in outcomes (28). Furthermore, it was found that sex abuse exhibits the highest degree of synergistic reactivity. Overall, the cumulative burden of chronic stress resulting from exposure to ACEs and subsequent NLEs, heightens the risk of allostatic overload and adversely affects both physical and mental health outcomes (29).

However, there is a scarcity of research investigating the prevalence, types, and combinations of different “non-medical” problems among those receiving work disability benefits (30). Moreover, even when life experiences are common, the context, perception, and consequences of these experiences can differ significantly among individuals. But, there are few studies on life experiences and health that adopt a descriptive in-depth approach (31). As a result, data on personal interpretations are scarce, and available information is less comprehensive. To truly grasp the importance of life events on health disorders, it is vital to understand how an individual's personal history shapes these experiences and influences their reactions to current life events (32). According to Berens and colleagues, there is also a need for increased clinical screening, scale-up of effective interventions, and research to develop new evidence-based response strategies to reduce lifetime risk of adverse outcomes among those exposed to adverse exposures early in life (33). This highlights a significant gap in our understanding that warrants further investigation.

The health challenges among patients referred to occupational rehabilitation are characterized by a multitude of symptoms, especially musculoskeletal and mental health complaints, and comorbidities (30, 34–37). Their employment history are also marked by frequent transitions in and out of the workforce both before and after rehabilitation (38). However, the prevalence of ACEs or NLEs and their possible impact on health has not been explored. Given the relatively unexplored nature of this field, we have chosen a mixed-method approach. This methodology allows us to gain a holistic understanding of the prevalence of negative life events and their association with health, while also incorporating patients’ personal experiences and perceptions of these events’ impacts on their health. By doing so, we achieve a more nuanced and context-specific understanding of the complex issues within the patient sample, exceeding what could be realized through either quantitative or qualitative methods in isolation (39). The objectives of this study are as follows:

• Demographic, Health, and Sickness Absence Characteristics: To examine the demographic profiles, health status, and patterns of sickness absence among the study participants.
• Prevalence and Subjective Experiences of ACEs: To determine the prevalence ACEs among the participants and explore their subjective experiences related to these events.
• Prevalence and Subjective Experiences of NLEs: To assess the prevalence of NLEs in the context of work and private life among the participants and delve into their personal experiences of these events.
• Comparative Analysis of ACEs Exposure: To investigate the differences between participants who have experienced ACEs and those who have not, particularly in terms of exposure to later negative events and their impact on health. This also includes identifying potential subgroups of ACEs that may have more troubled experiences.

## Materials and methods

### Design and setting

A convergent parallel design was applied in this descriptive mixed-methods study to get more in-depth knowledge and contextual understanding of the complexity issues in the patient sample (40). That is, we collected data from a survey and in-depth interviews concurrently, initially explored the data separately to get an overview, and then compared and integrated the findings. Integration of data and analysis occurred in an incremental way, moving back and forth between the quantitative and qualitative data and by use of inductive and deductive methods. The philosophical foundation for the study is pragmatism influenced by a critical realist perspective (41, 42).

This study is part of a larger study (the STAIR project) aiming to evaluate differences in the return-to-work rate between two groups of patients participating in a 4-week inpatient program or a 3-month outpatient program in Norway (43). Both clinics deliver complex occupational rehabilitation in the specialist health care. In the current study, data from the first part of the project, which was a pilot randomized controlled trial (RCT), was used to describe patient complexity. This is the first time the data from the study has been presented. The study is registered at current controlled trials: http://www.isrctn.com/ISRCTN12033424.

### Context

In Norway, employees suffering from musculoskeletal disorders and common mental disorders typically consult a general practitioner to obtain a sick leave note. The practitioner may then either provide follow-up care directly, refer the patient to for example a physiotherapist or psychologist in primary care, or recommend an occupational rehabilitation program within the specialist healthcare system as is the case for the patients in this study.

According to the National Insurance Act, all employees are entitled to sickness benefits if they are unable to work due to disease, illness, or injury (44). Sickness benefits are paid from the first day of absence for a period of up to 52 weeks, usually at the same level as employment income. Following the sick leave period, work assessment allowances or disability pensions may be granted if work ability is reduced by 50% or more. These allowances are 2/3 or less of the employee's income.

### Participants

The study recruited patients referred to either of the two participating clinics. Inclusion criteria: Employees with musculoskeletal disorders (MSDs), common mental disorders (CMDs), or a combination. They may also have additional disorders. Those included had to be on sick leave for at least 6 weeks, either in one continues period or several periods during the last 12 months; age 18–60 years; sufficient Norwegian language skills to fill out questionnaires. Exclusion criteria: severe psychological disorders (e.g., schizophrenia and other psychotic disorders, bipolar disorder, or personality disorders); substance addiction; pregnancy; more than two years out of work. The screening was made by the clinics.

The participants are representative for patients referred to the participating clinics regarding gender and age. Among 597 patients at clinic 1 in 2018, 71% were women, with a mean age of 47. Among 164 patients attending clinic 2 in the period 2013–2018, 79% were women, with a mean age of 44. In this study, 76% of the participants were women with a mean age of 44. Additionally, as for the study participants, all patients referred to the two occupational rehabilitation clinics have musculoskeletal complaints and/or common mental complaints. The health problems of this patient group are in accordance with what is described in other Norwegian studies among patients referred to occupational rehabilitation (36, 45, 46).

### Recruitment

All eligible patients referred to the clinics were invited to participate in the study through written and oral information about the study just before arrival or at arrival. Recruitment and collection of consent forms were organized by the clinics during the first week of the program. The first participant was recruited in April 2014, and the last in November 2017 (*N* = 80). When we reached 40 participants, we began recruiting participants for interviews. A total of 39 participants agreed to participate in interviews, the first was recruited in April 2015. After being included in the study, participants were randomly assigned to one of the clinics.

For more detailed information on study setting and interventions, see Eftedal and colleagues (43).

### Measures and assessments

#### Quantitative data collection

In the quantitative strand of the study, data was collected through a survey. The participants received an electronic baseline survey covering demographics, work, health complaints, and sick leave. In addition, we asked 19 different questions about negative life events during lifetime, 9 ACEs and 10 NLEs (see Table 1).

**Table 1:** Distribution of negative life events across subgroups of participants based on their history of childhood and adult negative life events.

Adverse childhood experiences	Group 1 *n* = 34	Group 2 *n* = 26	Group 3 *n* = 20	Total	*p*-value
	Number (%)	Number (%)	Number (%)	Number (%)	
Did you grow up with parents/guardians who					
Abused alcohol or other drugs?					<0.001
No	34 (100)	22 (88)	12 (60)	68 (86)	
Yes	0 (0)	3 (12)	8 (40)	11 (14)	
Were mentally ill or attempted suicide?					<0.05
No	34 (100)	25 (96)	17 (85)	76 (95)	
Yes	0 (0)	1 (4)	3 (15)	4 (5)	
Did you experience parents or other adult subjected you to:					
Physical violence (e.g., hitting or pushing)?					<0.001
No	34 (100)	20 (77)	10 (53)	64 (81)	
Yes	0 (0)	6 (23)	9 (47)	15 (19)	
Psychological violence (e.g., curses or humiliations)?					<0.001
No	34 (100)	19 (76)	6 (32)	59 (76)	
Yes	0 (0)	6 (24)	13 (68)	19 (24)	
Sexual abuse?					<0.005
No	34 (100)	19 (73)	14 (74)	67 (85)	
Yes	0 (0)	7 (27)	5 (26)	12 (15)	
Did you often or very often experience as a child:					
Feeling worthless or unloved?					<0.001
No	34 (100)	13 (50)	8 (40)	55 (69)	
Yes	0 (0)	13 (50)	12 (60)	25 (31)	
Being abandoned or left to fend for yourself without care or supervision?					<0.01
No	34 (100)	24 (96)	16 (80)	74 (94)	
Yes	0 (0)	1 (4)	4 (20)	5 (6)	
Negative life events in adult life					
Have you experienced any of the following during your lifetime?					
Death of a child, spouse/partner?					<0.05
No	32 (97)	21 (84)	14 (74)	67 (87)	
Yes, in the last 12 months	0 (0)	2 (8)	0 (0)	1 (3)	
Yes, in the past	1 (3)	2 (8)	5 (26)	8 (10)	
Death of other close family members?					0.530
No	4 (12)	6 (23)	2 (10)	12 (15)	
Yes, in the last 12 months	4 (12)	1 (4)	1 (5)	6 (8)	
Yes, in the past	26 (77)	19 (73)	16 (84)	61 (77)	
Experienced serious illness or injury among your close ones?					0.550
No	9 (27)	9 (35)	6 (30)	24 (30)	
Yes, in the last 12 months	7 (21)	3 (12)	1 (5)	11 (14)	
Yes, in the past	18 (53)	14 (54)	13 (65)	45 (56)	
Experienced severe financial problems?					<0.001
No	26 (7)	12 (46)	7 (35)	45 (56)	
Yes, in the last 12 months	3 (9)	8 (31)	0 (0)	11 (14)	
Yes, in the past	5 (15)	6 (23)	13 (65)	24 (30)	
Experienced problems or conflicts in close relationships (family, friends)?					<0.001
No	26 (79)	9 (35)	5 (25)	40 (51)	
Yes, in the last 12 months	3 (9)	5 (19)	2 (10)	10 (13)	
Yes, in the past	4 (12)	12 (46)	13 (65)	29 (37)	
Experienced marital or long-term relationship breakdown?					<0.004
No	20 (59)	8 (31)	5 (25)	33 (41)	
Yes, in the last 12 months	0 (0)	4 (15)	0 (0)	4 (5)	
Yes, in the past	14 (41)	14 (54)	15 (75)	43 (54)	
Been subjected to bullying at work or school?					<0.001
No	31 (94)	17 (65)	6 (30)	54 (69)	
Yes, in the last 12 months	2 (6)	1 (4)	0 (0)	3 (4)	
Yes, in the past	0 (0)	8 (31)	14 (70)	22 (28)	
Have you been unemployed?					0.160
No	24 (71)	15 (60)	8 (40)	47 (60)	
Yes, in the last 12 months	5 (15)	2 (8)	4 (20)	11 (14)	
Yes, in the past	5 (15)	8 (32)	8 (40)	21 (27)	
Been subjected to violence or abuse (physical or psychological) after the age of 18?					<0.001
No	28 (82)	23 (89)	8 (40)	59 (74)	
Yes, in the last 12 months	0 (0)	0 (0)	2 (10)	2 (3)	
Yes, in the past	6 (18)	3 (12)	10 (50)	19 (24)	
Have you been in imminent danger of loss of life due to a serious accident, disaster, violent situation, or war?					0.480
No	32 (94)	22 (85)	18 (90)	72 (90)	
Yes, in the last 12 months					
Yes, in the past	2 (6)	4 (15)	2 (10)	8 (10)	

Two items have been excluded from the Adverse Childhood Experiences questionnaire in the table because they were either not experienced by the participants or were reported by only one person: “*Did you grow up with parents or guardians who were convicted or imprisoned?*” *and* “*Did you experience often or very often as a child to become neglected by not receiving enough food, clean and warm clothes, or by not being taken to a doctor when required?*”*.*

Group 1: participants without adverse childhood experiences.

Group 2: participants with adverse childhood experiences, but not revictimized.

Group 3: participants with adverse childhood experiences and revictimized.

The difference between groups was evaluated using table analysis and the Pearson chi-square test, indicated by the *p*-value. The mean number of life events reported in childhood was 1.5 and 2.7 for Groups 2 and 3, respectively, out of a maximum of 9 events. In adulthood, the mean number of life events reported was 3.1, 4.5 and 5.9 for the Groups 1, 2 and 3, respectively, out of a maximum of 10 events. The differences across all groups are statistically significant, with *p*-values ranging from less than 0.01 to less than 0.001.

## ACE

We utilized a modified version of the ACEs-questionnaire, originally developed by Felitti et al. in 1998). The modification was necessitated by the ethical committee's concerns regarding the sensitivity of the original questionnaire's content. The revised set of questions was crafted by Anna Luise Kirkengen, an experienced practitioner and researcher in this field. Three questions probe whether the participants grew up with parents who themselves had problems; three questions probe exposure to violence in childhood by adults or parents; and three questions probe neglect (for full list of items see Table 1). Response options were “yes” and “no”. Cronbach's alpha based on our sample is 0.70.

## NLE

The 10 questions about other NLEs included: conflicts in close relations; serious financial problems; experience of losing your job; having been exposed to bullying at school or workplace; violence, or abuse after the age of 18 etc. (for full list of items see Table 1). Response options were “occurred during the last 12 months”, “occurred prior to the last 12 months” or “not at all”. This is mainly a collection of questions used in different surveys on living conditions by Statistics of Norway and HUNT3, the Nord-Trøndelag Health study. Clinicians and researchers working in occupational rehabilitation, who had insight into stressful life-events among their patients, were involved in the selection of questions. The response alternatives were decided by the project group. Cronbach's alpha based on our sample is 0.56.

## Health

Health complaints were probed with the Subjective Health Complaints Inventory (SHC), which is validated on a Norwegian population (47) and Hospital Anxiety and Depression Scale (HADS) (48), also validated on a Norwegian population (49). The SHC inventory consists of twenty-nine questions concerning subjective and somatic health scored on a four-point scale from zero (no complaints) to three (serious complaints), with a minimum sum score of 0 and maximum sum score of 87. The SHC inventory yields scores on single items and a total number of health complaints categorized into five factors: musculoskeletal pain, pseudoneurology, gastrointestinal problems, allergy, and flu. However, in this study we have only used scores on single items.

The HADS scale consists of seven items for anxiety and seven for depression, each scored on a four-point scale from zero (not present) to three (considerable). Sum scores for each of the scales range from a minimum of 0 to a maximum of 21. A score above 11 are considered indicative of anxiety or depression in need of further investigation and possibly treatment. A score from 8 to 10 is considered a possible case. The total sum score for both the scales is 42. Sum scores were calculated for each of the anxiety and depression scales, in addition to a total score. Cronbach's alpha based on our sample is 0.86.

To investigate whether the participants viewed life events as a significant cause of distress, we utilized the following question: “What impact have the event(s) had on your health?” on a four-point scale from “not at all” to “in high degree”. We also used three open-ended questions from the Illness Perception Questionnaire (50). The respondents were asked to “rank the three most important factors you believe are the cause of your illness/troubles”.

### Qualitative data collection

In the qualitative strand of the study, in-depth face-to-face interviews were used, during the first week of rehabilitation applying a semi-structured interview guide with open-ended questions. A project group, consisting of researchers and occupational rehabilitation clinicians, developed the interview guide. This was subsequently refined based on insights gained from the initial informant interviews. The intention of this interview guide was to understand the patients’ health problems, their experiences of their own situation, and the reasons for their health problems. Additionally, it aimed to understand their context regarding private life and work, which might inhibit and promote their process back to work.

The informants were first asked to provide information about themselves and the reason for being referred to occupational rehabilitation. Follow-up probes were asked on the following topics: (a) Family obligations, (b) Work, education/profession, possibly other previous jobs, (c) Current health situation, including symptoms and use of medication, (d) Duration and reason of sick leave, (e) Receipt of benefits, (f) Own thoughts about the causes of symptoms. They were then asked to elaborate on the consequences of these issues for them, with follow-up probes on private and work-related impacts. Additionally, they were asked to describe their perceived work ability in relation to their job demands, with probes on physical and mental demands, self-management/control, social support, confidence in their ability to meet job requirements, and their expectations for RTW. Informants who had experienced ACEs or other NLEs reported in the questionnaire were asked if they wanted to talk about these incidents but were also told they could refrain from elaborating. Each interview lasted approximately 1 h and was audio-recorded and transcribed verbatim.

#### Data analysis

The processes of mixed-methods analysis described by Pat Bazeley were followed, which is an iterative process in seven steps where conversation between interviews and survey responses is used to identify concepts and themes (39). Qualitative data were analyzed using the reflexive thematic analysis approach described by Braun and Clark (51–53). Comparative analyses were made to answer research questions, comparing across subgroups, and investigating patterns of association. The data were integrated by combining information from tables and narratives and by going back and forth between the qualitative and the quantitative data-material during the analysis. NVivo 1.7. were used in the analysis of the qualitative data and SPSS version 28.01.0 was employed for the quantitative analyses.

The quality and validity of the quantitative and qualitative data was checked in the preparation for the analysis to increase the rigor of the data. The researchers worked as a transdisciplinary team to ensure trustworthiness of the data. In the qualitative analyses we worked in circulating pairs when extracting text and making summaries. Our different backgrounds from physiotherapy and sociology (ME), nursing and epidemiology (RK), and psychology (TJ) ensured we extracted different elements from the text and brought in different perspectives to the discussions. All the codes were re-read and checked for completeness in the different phases by at least two researchers. The integration of data involved an iterative process of navigating between qualitative and quantitative datasets. This method ensured alignment in the questions posed to both types of data, facilitating synthesis to enhance the overall comprehensiveness of the dataset.

### Quantitative analysis

Independent samples *t*-tests were conducted on continuous data to evaluate whether there was a statistical difference between individuals with a history of ACEs (Groups 2 and 3) and those without a history of ACEs (Group 1). A two-tailed test with an alpha of 0.05% and 95% confidence intervals was used. Categorical data were summarized in tables, and Chi-square tests were conducted to investigate if there were significant differences between the groups. We considered results statistically significant if the *p*-value was less than 0.05, with two degrees of freedom.

### Power analysis

*Post hoc* power calculations using G*Power (54) were performed to check the power of the quantitative results which were significant. Results indicated that with an average effects size based on the significant results of 0.33, a two-tailed alpha level of 0.05, total sample size of 80 and number of groups at three produced a power of 0.74.

### Description of the analytical process

1. *In phase one,* we prepared the quantitative and qualitative data for analysis. All three members of the research team read six transcripts to obtain an overall impression of the qualitative data to understand the complexity of the informants regarding their situation and context. Then we discussed our impressions and agreed on a template for extracting information from the transcripts. The templates were organized in chapters covering the following topics: history of sickness absence and benefits; demographics; family; employment history; health history and symptoms; stressful life experiences; the informants’ thoughts about causes of symptoms. In the following we decided to work in circulating pairs. One person read the transcript, extracted text that fitted with the topics and wrote a summary of the transcript. The second person read through the memo and the transcript and added additional information when needed.
2. *In phase two*, initial descriptive statistics were made to get an overview of the quantitative data and to check for any gaps and coding errors. In the qualitative data, all written memos were coded at the topic level. All three researchers noted their reflections and thoughts on coding and preliminary themes in personal memos. We then agreed on some preliminary codes in phase three. These codes were based on existing concepts.
3. *In phase three,* we read through each topic to refine codes and identify possible themes. In this phase the codes used are a mix of long empirical codes that is closely related to what the informants say (semantic), and short codes that encompass our conceptual and theoretical framework. When needed we went back to the full condensed text on each informant to capture information that was relevant to our codes that cut across chapter topics, also ensuring that important context information was not missed. When needed, we also went back to the full transcripts and coded directly there. We also compared information from questionnaire and interview for each of the participants to fill out any missing information in either of them regarding background information or experiences of life events.
4. *In phase four,* we examine the codes and collated data to identify significant broader patterns of meaning and generate initial subcodes. The sub-codes found for life experiences in childhood and adulthood among the informants are mainly in line with the variables in the questionnaire used at baseline. In addition, we found some additional subcodes in the qualitative material that affected health, such as: “complications related to surgery”, and “demanding care for children and parents” in the private context.

In this phase, we also specifically looked for any subgroups of participants based on differences in life events in childhood and adulthood. We first conducted in-depth analyses of the interviews with each individual informant, supported by the development of codes, concept maps, case attributes, and crosstabulation queries in NVivo. By combining information from the qualitative and quantitative data, we discovered that those with a history of violence in childhood and experiences of new victimization in adolescence or adulthood seemed to be the most exposed to NLEs during lifetime. Also, not part of the ACEs items, experiences of bullying as a child or adolescent were important for the informants’ health. We decided to split those who had experienced ACEs in two groups based on whether they were revictimized or not, including experiences of bullying and violence in in adolescence or adulthood, and compare with those without reporting on ACEs. The classifications of groups were Group (1) Those without ACEs; Group (2) Those with ACEs but not revictimized; Group (3) Those revictimized.

Revictimized individuals are defined as:

• Physical/psychological violence or sexual abuse as children, and
• Experience of bullying, and/or
• Physical/psychological violence or abuse after 18 years

Those who had not experienced ACEs but had been subjected to bullying earlier were placed in Group 2 (6p). One person without ACEs but with experiences of early bullying and violence/abuse after the age of 18 was categorized as revictimized. However, since we are not able to separate bullying in school and workplace in the questionnaire among those not interviewed, it is possible that 3 without ACEs are misclassified as belonging to Group 2 (2persons) or Group 3(1person), instead of Group 1. The results were subsequently validated through identification of relevant literature and by creating corresponding subgroups both as an attribute in NVivo and variable in SPSS. These groupings were then tested using table analyses in both NVivo and SPSS, in addition to *t*-tests in SPSS.

5. *In phase five*, we reviewed and rearranged codes, clustered some together, and formulated the codes as themes. Examples of identified themes impacting the participants’ health in the context of private life were: “Trouble in private relations due to conflicts, violence and harassment”, “Other relational strains in the family”, “A strained economy is a source of stress and worries”. In the work context, work-related sub-codes identified were “a psychological demanding work situation due to workload, conflicts, feelings of incompetence or uncertainty”, “A long history of a physical demanding jobs and work accidents influencing physical health”. We also checked the themes against the dataset again to ensure that we had captured the most important themes in relation to the research questions. Patterns of responses were compared across subgroups and across quantitative and qualitative material to further refine the descriptions of the data.
6. *In phase six*, relationships between variables are explored further for more details. Qualitative analysis of an open question in the survey (“rank the three most important factors you believe are the cause of your illness/troubles”) was also done looking for possible life experiences affecting health contradicting or supporting our findings from the interviews. Then the last quantitative analysis of associations in the material were made before the final decision on which themes to include. Alternative models of subgroups were also tested out, i.e., we compared differences in the impact on health in table analysis in SPSS by use of the three subgroups with and without ACEs exposure and revictimization, and by different groupings of number of ACEs.
7. *In the final phase,* the findings were written, and the analysis contextualized in relation to existing literature.

#### Ethical approval and consent to participate

The research was carried out in compliance with the principles of the Declaration of Helsinki. The study was approved by the Regional Committee for Medical and Health Research Ethics in Western Norway (2011/934). The participants provided a written informed consent to participate and indicated whether they wanted to be contacted for interviews. They were informed about their participation being voluntary, that they had the right to withdraw at any time without having to state a reason, and that the reporting from the study would use anonymized data.

## Results

In the presentation of results, we have chosen to integrate the findings from the complementary qualitative and quantitative material to compose a more coherent picture, starting with the characteristics of the participants. Then follow the prevalence of negative life experiences illustrated with quotes from the qualitative material. In the last part we present the participants’ and informants’ views of obstacles they perceive as the cause of their health complaints.

In the following text we use “participants” when referring to the whole sample, “respondents” when talking about survey results and “informants” when talking about results from interviews.

### Characteristics of the participants

*Demographic and socioeconomic:* Descriptive information about the study participants in total (*n* = 80), and the subsample participating in interviews (*n* = 39), is provided in Table 2. Most participants were females (76%) with a mean age of 45 years. The majority were married (67%) and had children (79%). Regarding educational level, 25% had low level, while 39% and 36% of the participants had medium or high level respectively. Most of the participants were employed (83%) and worked in service and manual occupations such as healthcare services (27%), office-based services (26%), retail (17%), and others such as kindergartens/school, workshops, and driving. Many of the participants encountered financial strains (60%), while 25% experienced more severe financial challenges. Nearly all participants received health-related benefits of which 67% received sick leave benefits while 25% received work assessment allowance (i.e., one year or more on health-related benefits). There were no significant differences on the demographic and socioeconomic variables between those who were interviewed and those who were not (tested with table analysis and the Pearson chi-square test).

**Table 2:** Distribution of demographic and health characteristics of the study sample.

Socioeconomic variables	Not interviewed (1) *N* = 41	Interviewed (2) *N* = 39	Total *N* = 80	Difference (1, 2)
	Number (%)	Number (%)	Number (%)	Significance
Sex				ns
Male	11 (27)	8 (21)	19 (24)	
Female	30 (73)	31 (80)	61 (76)	
Age				ns
<40	11 (27)	8 (21)	19 (24)	
40–49	21 (51)	21 (54)	42 (53)	
50<	9 (22)	10 (26)	19 (24)	
Civil status^a^				ns
Cohabitant	30 (73)	24 (62)	54 (68)	
Not cohabiting	11 (27)	15 (39)	26 (33)	
Number of children				ns
0	8 (20)	9 (23)	17 (21)	
1	6 (15)	10 (26)	16 (20)	
2	19 (46)	10 (26)	29 (36)	
3+	8 (20)	10 (26)	18 (23)	
Educational level^b^				ns
Low	11 (27)	9 (23)	20 (25)	
Medium	16 (39)	15 (39)	31 (39)	
High	14 (34)	15 (39)	29 (36)	
Occupation				ns
Healthcare personnel, low-skilled	3 (7)	7 (18)	10 (13)	
Healthcare personnel, highly skilled	5 (12)	7 (18)	12 (15)	
Kindergarten/preschool/School	3 (7)	4 (10)	7 (9)	
Store/retail	10 (24)	4 (10)	14 (18)	
Office	14 (34)	7 (18)	21 (26)	
Workshop	4 (10)	2 (5)	6 (8)	
Other/various^c^	2 (5)	8 (21)	10 (13)	
Paid work				ns
No	8 (20)	10 (26)	18 (23)	
Yes	33 (81)	29 (74)	62 (78)	
Position size				
<50%	5 (15)	3 (9)	8 (12)	
50%–90%	7 (21)	10 (30)	17 (26)	
>90%	21 (64)	20 (61)	41 (62)	
Self-assessed family finances				ns
Unstrained economy	15 (37)	17 (44)	32 (40)	
Some strains in economy	17 (42)	11 (28)	28 (35)	
Strained economy	9 (22)	11 (28)	20 (25)	
Social benefits				ns
Sick leave				
Part-time	12 (29)	10 (26)	22 (28)	
Full-time	16 (39)	16 (41)	32 (40)	
Work assessment allowance				
Part-time	4 (10)	5 (13)	9 (11)	
Full-time	4 (10)	7 (18)	11 (14)	
Disability pension				
Part-time	4 (10)	1	5 (6)	

The difference between groups was evaluated using table analysis and the Pearson chi-square test. All tests were above *p* = 0.05, indicated as not significant (ns).

^a^ Civil status: cohabitant includes married and partnership. Not cohabiting include single, divorced, separated, and widowed.

^b^ Educational level: low includes primary and secondary education without a vocational certificate. Medium includes Upper secondary education with specialization in general studies or vocational certificate. High includes college/university education of 3 years or more.

^c^ Other/various includes drivers, transport workers, cleaners, technicians, cook, facilities managers, craftsmen.

*Subgroups:* During the analyses we looked specifically for any subgroups of participants based on their history of life events in childhood and adulthood. By combining information from the qualitative and quantitative data, we discovered that those with a history of violence or abuse in childhood and experiences of new victimization in adolescence or adulthood seemed to be the most exposed to NLEs during lifetime. Based on this, we decided to divide those who had experienced ACEs into two groups, and compare with those without any reporting on ACEs:

Group 1: Those without ACEs; Group 2: Those with ACEs but not revictimized; Group 3: Those with ACEs and revictimized. We categorized participants as revictimized if they reported physical violence, psychological violence, or sexual abuse during childhood, coupled with experiences of bullying prior to the last 12 months, and/or physical/psychological violence or abuse occurring after the age of 18 years. A total of 25% of the participants were categorized as revictimized. For more details, see phase 4 in the method section.

**Figure A1:**
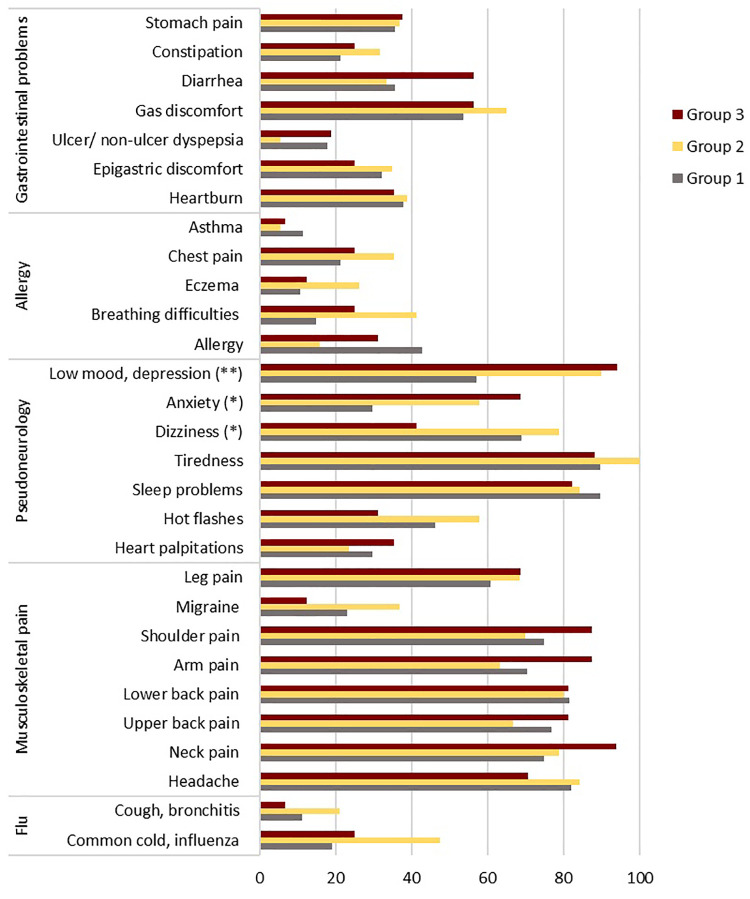
The total of subjective health complaints among study participants stratified by their history of childhood and adult negative life events. Group 1: participants without adverse childhood experiences (*n* = 34). Group 2: participants with adverse childhood experiences, but not revictimized (*n* = 26). Group 3: participants with adverse childhood experiences and revictimized (*n* = 20). Differences between groups evaluated by chi-square test (*p < 0.05, **p < 0.01, ***p < 0.001). Scale used: 0 = No complaints, 1 = Any complaints (1–3).

*Health:* The respondents and informants reported an average of 14 health symptoms each (the possible maximum of health symptoms was 29). An overview of health problems from the SHC is presented in Figure A1. The most common reported health symptoms were depression, anxiety, tiredness, sleep problems and muscular pain. Many also reported gastrointestinal issues.

**Figure 1:**
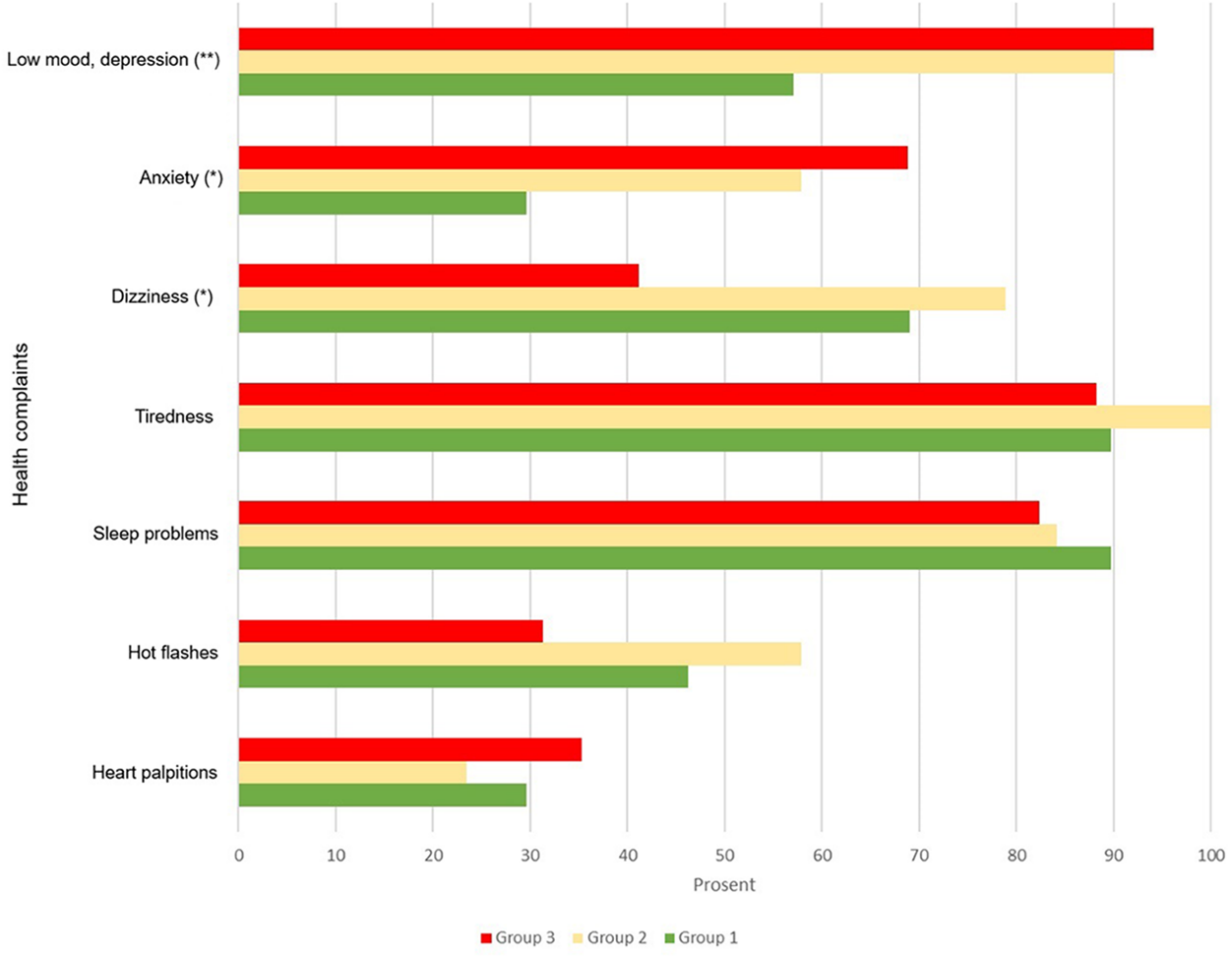
Subjective health complaints among study participants stratified by their history of childhood and adult negative life events. Group 1: participants without adverse childhood experiences (*n* = 34). Group 2: participants with adverse childhood experiences, but not revictimized (*n* = 26). Group 3: participants with adverse childhood experiences and revictimized (*n* = 20). Differences between groups evaluated by chi-square test (*p < 0.05, **p < 0.01, ***p < 0.001). Scale used: 0 = No complaints, 1 = Any complaints (1–3).

Figure 1 shows pseudoneurology complaints from the subjective health complaints inventory among participants across subgroups based on their history of childhood and adult negative life events. Pseudoneurology or “medically unexplained” complaints are the name given for symptoms which appear to be caused by problems in the nervous system, but which are not caused by a physical neurological disease or disorder. Comparison between groups showed that individuals with a history of ACEs (Groups 2 and 3) reported more symptoms of anxiety (%-diff. = 40, *p* < 0.01) and depression compared to Group 1 (%-diff. = 39−32, *p* < 0.01) (see Figure 1). However, no differences were observed concerning other health issues. Analyzing sum-scores for HADs across groups, we observed that Group 2 experienced significantly more symptoms of anxiety and depression than Group 1 (*p* = 0.01). The percentage difference among those reporting a score of 8 or more is 40% for anxiety and 33% for depression. Group 3 was also overrepresented in terms of symptoms of anxiety compared to Group 1 (%-diff = 42, *p* = 0.02), but the difference between groups was less regarding symptoms of depression (%-diff = 35, *p* = 0.09) (see Table 3). No differences were found concerning other health complaints such as musculoskeletal complaints (Figure A1).

**Table 3:** Depression and anxiety across subgroups of participants based on their history of childhood and adult negative life events.

HADS	Group 1 (*n* = 34)	Group 2 (*n* = 26)	*t*-value (G2 vs. 1)	95% CI (G2 vs. 1)	Group 3 (*N* = 20)	*t*-value (G3 vs. 1)	95% CI (G3 vs. 1)
HADS—total							
Mean (SD)	12.1 (7.1)	18.0 (5.4)	3.5***	2.6–9.3	16.8 (6.7)	2.4*	0.8–8.6
Anxiety							
Mean (SD)	6.6 (4.5)	9.4 (3.3)	2.6***	0.7–4.8	9.5 (3.6)	2.4*	0.5–5.3
0–7	20 (59%)	7 (27%)			6 (30%)		
8–10	4 (12%)	8 (31%)			6 (30%)		
11≥	10 (29%)	11 (38%)			8 (43%)		
Depression							
Mean (SD)	5.4 (3.8)	8.6 (3.1)	3.5***	1.3–5.0	7.3 (3.9)	1.7	−0.3 to 4.0
0–7	27 (79%)	11 (42%)			10 (50%)		
8–10	2 (6%)	8 (31%)			6 (30%)		
3 (11≥)	5 (15%)	7 (27%)			4 (20%)		

Group 1: participants without adverse childhood experiences.

Group 2: participants with adverse childhood experiences, but not revictimized.

Group 3: participants with adverse childhood experiences and revictimized.

The table shows the percentage distribution of symptom pressure for anxiety and depression. Sum scores for each of the scales range from a minimum of 0 to a maximum of 21. A score above 11 are considered indicative of anxiety or depression in need of further investigation and possibly treatment. A score from 8 to 10 is considered a possible case. The total sum score for both the scales is 42.

Differences in the mean number of symptoms for HADs total, anxiety and depression between individuals without a history of negative life events in childhood (Group 1) and individuals with a history of negative life events in childhood (Groups 2 and 3) were evaluated using an independent sample *t*-test. There were no significant differences between Groups 2 and 3 in terms of symptoms of anxiety and depression.

* *p* ≤ 0.05.

*** *p* < 0.001.

In the interviews, many informants described how their physical and mental burdens resulted in bodily unexplained and diffuse symptoms and pains that varied over time, rumination, sleep problems and fatigue. Several were diagnosed with fibromyalgia, some with rheumatic diseases, burnout, and post-traumatic stress. Some also described experiences of hormonal dysfunctions, and various infections. A few talked about having had or having suicidal thoughts.

Most of the informants reported long-term symptoms and having been in transition between sickness absence and work on several occasions. Some of them had reduced their working hours to be able to stay at work.

#### Negative life events

The prevalence of negative life events (ACEs and NLEs) across groups are presented in Table 1.

### Adverse childhood experiences

About half (49%) of the participants reported at least one ACEs from childhood. Of these, 18% reported one ACE, 22% reported 2−3 ACEs and 9% reported 4 or more ACEs.

The most common ACEs reported by the participants were experiences of not being loved (neglect) (31%), psychological and physical violence (24% and 19% respectively) and sexual abuse (15%).

There were significant differences across the three identified groups (see Table 1). Comparison between the two ACEs groups, indicated that Group 3, characterized by revictimization, exhibited the highest degree of exposure to various ACEs, with a mean of 2.7 events per individual. Within this group, 60% of the participants reported feelings of not being loved as a child, 68% reported psychological violence, and nearly half reported exposure to physical violence. Individuals in Group 2 reported similar challenges, albeit with lower prevalence than those observed in Group 3, except for sexual abuse that was reported by 26% in both groups. The observed differences between the 2 ACE groups were significant higher for Group 3 than 2 regarding exposure to psychological violence (%-diff = 44, *p* < 0.01) and parental drug misuse (%-diff = 28, *p* = 0.03). Additionally, non-significant statistical differences were noted in relation to exposure to physical violence (%-diff. = 24, *p* = 0.09) and instances of neglect (abandonment, %-diff. = 16, *p* = 0.09).

Participants who grew up with a parent struggling with substance abuse (14%) and/or mental illness (5%), reported heightened exposure to multiple ACEs. Moreover, this subgroup more frequently experienced psychological violence and neglect. The analysis did not reveal significant variations across demographic factors, with the exception that men to a greater extent than women reported being exposed to physical violence (37% vs. 13%, *p* = 0.02).

An example of the most common ACE reported by participants, feelings of not being loved, is the case of an informant in Group 2 who grew up in a family with an alcoholic father and a mentally ill mother:

“I've never really had, um, experienced that anyone cared about me, my dad died early, and then I had a grandmother, um, we lived in the same house and she, she was the one who noticed and took care of me, so to speak.” Group 2, nr. 1, female

An informant who reported both physical and psychological violence in childhood described his mother's behavior in this way:

“She could be … yeah, could suddenly get angry and maybe give us a slap in the face and threaten us in various ways. It wasn't always that we were wanted, ha-ha, you could say.” Group 2, nr. 2, male

Another informant about his violent father:

“You got a pat on the back when you did a good job and beaten when you did poorly. And then you had to go out into the woods and get your own birch branches.” Group 3, nr. 1, male

Both female and male informants had experienced sexual abuse when they grew up. One of the revictimized male informants had experienced sexual abuse by his biological father since he was one and a half years old. He had kept it as a secret to himself in all these years, until he responded on the survey:

“I don't have any contact with my biological father because of some things that happened throughout my childhood that I will never forgive him for… I would never let my children be around him without supervision. … (if my new father) had known about the things I'm struggling with, he would have gone and killed him—I'm sure of it. So maybe I should have said something. But I've always been that way, I've kept things to myself.” Group 3, nr. 2, male

A female informant has chosen to be more open about her experiences:

“I usually say that I have been a bit abused by my father. … incest is so difficult. … He is also a very authoritarian person whom I have chosen to exclude from my life.” Group 2, nr. 3, female

### Negative life events in adulthood and the accumulated burden of events during lifetime

**Figure 2:**
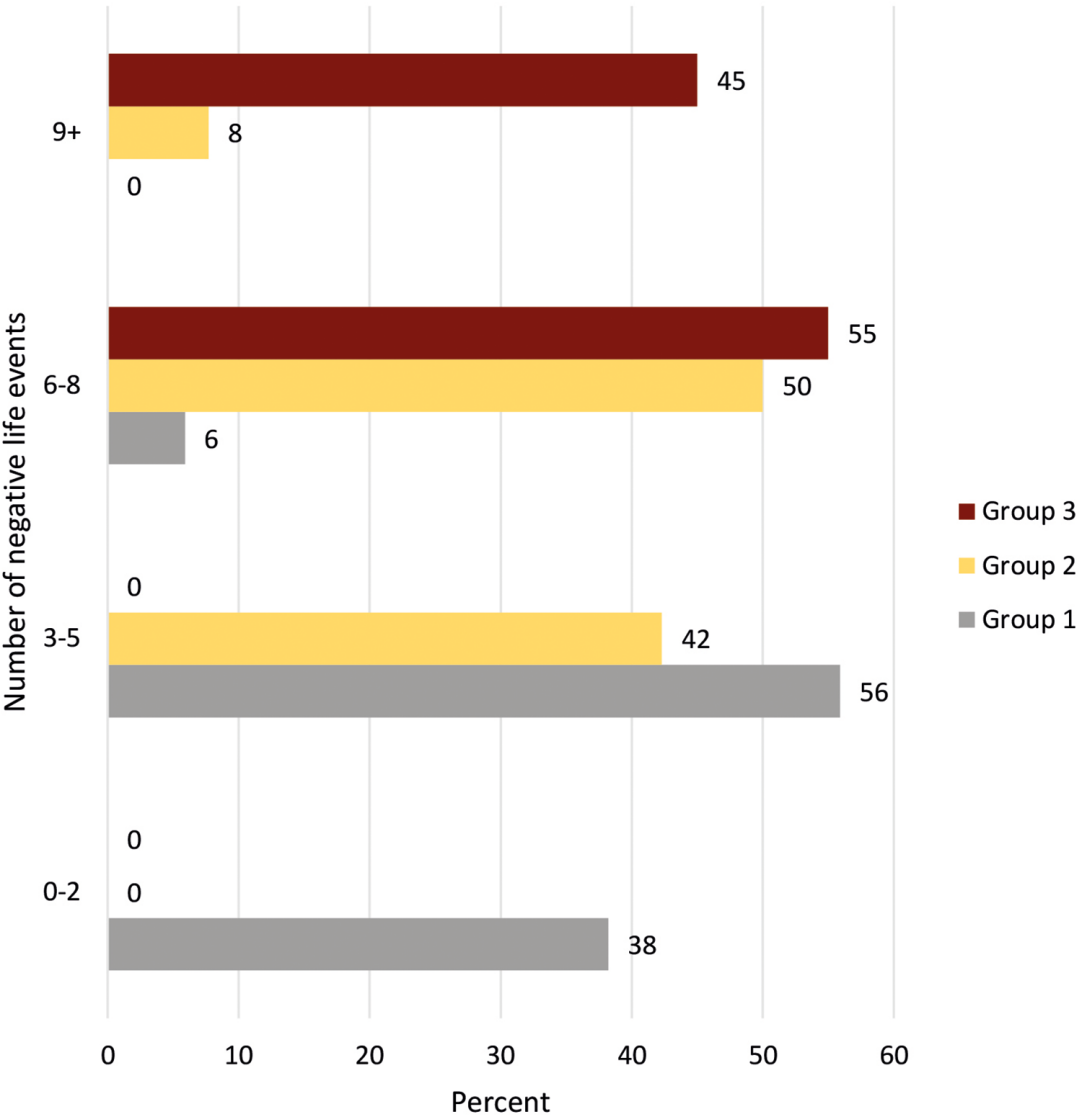
Number and percent of negative life experiences across subgroups of participants based on their history of childhood and adult negative life events. Group 1: participants without adverse childhood experiences (*n* = 34). Group 2: participants with adverse childhood experiences, but not revictimized (*n* = 26). Group 3: participants with adverse childhood experiences and revictimized (*n* = 20).

In addition to ACEs, the respondents could report up to 10 additional NLEs (see Table 1). The most common NLEs among the participants were death and serious illness among those close to them. Following that 59% reported experiences of break-ups, 50% reported conflict in close relations, and 44% reported serious financial problems. Also, 33% reported being subjected to violence or abuse during adulthood. Among those who had experienced any form of violence in childhood, 62% experienced new victimization in adulthood. The total number of NLEs reported in adulthood were higher than in childhood, with a mean of 4.2. When both ACEs and NLEs were summed, Group 3 reported a mean of 9 lifetime NLEs, with 45% reported 9 or more events and 55% reported 6–8 events. Group 2 reported a mean of 6 NLEs, where only 8% reported 9 or more events, 50% reported 6–8 events, and 42% reported 3–5 events. Group 1 reported a mean of 3 NLEs with 6% reporting 6–8 events, 56% reported 3–5 events, and 38% reported 1–2 events. The differences between all groups were tested with an independents sample *t*-test, all significant at *p* < 0.001. The number and percentage of lifetime NLEs distributed across groups is shown in Figure 2.

All individuals in Group 3 reported experiencing new incidents of victimization in adulthood (bullying and/or violence/abuse), whereas in Groups 2 and 1, 42% and 18%, respectively, had experienced victimization during adulthood. These differences were statistically significant (*p* < 0.001). Furthermore, individuals in Groups 2 and 3, reported significantly higher involvement in violence or conflicts within close relationships, and experiences of relationship breakdown, severe financial problems and death of a child or partner in adulthood compared to Group 1 (see Table 1). The disparities between Groups 2 and 3 are statistically significant concerning incidences of violence (*p* < 0.01), instances of bullying (*p* = 0.03), and financial challenges (*p* < 0.01), with Group 3 exhibiting the highest level of exposure. The total burden during lifetime is also highest in Group 3, but Group 2 have more of such experiences last year. Financial difficulties are predominantly reported by those who have gone through a relationship break-up compared to those without these experiences (57% vs. 24%, *p* < 0.01).

#### What significance do ACEs and NLEs have for the participants’ current health?

Nearly all the respondents reported that their history of NLEs had affected their health to some degree or to a high degree (77%), especially those with ACEs (90%). The analysis of the qualitative material supported and complemented the findings in the quantitative analyses (Figure 1), showing that the two groups with ACE exposures are struggling more with their mental health than those without ACE experiences.

To better understand the significance of ACEs and NLEs on the participants’ current health, we conducted an in-depth analysis using the informants perceptions of the causes of their health problems. We also incorporated qualitative analysis of the responses from the open-ended question in the questionnaire: “… describe and rank the three most important factors you believe are the cause of your complaints” as sources for the thematic analysis. The main themes identified are: Childhood and adolescent adversities may have a lasting negative impact on health; Several negative life experiences in the context of work are affecting health; Several negative life experiences in the private life are affecting health; and Complex interactions between negative life experiences and health (see Table 4).

**Table 4:** The significance of ACEs and NLEs on health—thematic analyses.

Themes	Subthemes
Childhood and adolescent adversities may have a lasting negative impact on health	• Early lifetime experiences such as ACEs and bullying may affect adult health in many ways • Exposure may increase vulnerability to new events affecting health in adulthood
Negative life experiences in the context of work that are affecting health	• Perceptions of work as too demanding—exceeding their work ability at the moment: ○ A psychological demanding work situation because of: ▪ too high workloads, ▪ relational problems and conflicts ▪ feelings of incompetence or uncertainty ▪ loss of job ○ Excessive physical strain due to many years in a physical demanding job with: ▪ heavy lifting ▪ monotone and repetitive work • Accidents at work causing health problems
Negative life experiences in the context of private life that are affecting health	• Perceptions of private life as too demanding due to: ○ Experiences of severe violence and harassment ○ Family-related burdens such as conflicts and breakups, substance abuse in close relations, caregiving due to sick children or parents, and deaths ○ Worries and stress due to a strained economy • Severe injuries from accidents or surgeries
Complex interactions between negative life experiences and health	• The total burden is too high ○ Living with multiple problems for many years ○ Many things occur at the same time in a short timeframe • Complexities of negative life experiences and the timespan involved make it difficult to understand the causes of their illnesses

### Childhood and adolescent adversities may have a lasting impact on health

This theme relates to the participants’ experiences and perceptions on how ACEs and bullying in childhood have affected their health both during youth and as adults. It also encompasses their opinions on whether early experiences have influenced their reactions to stressful situations in adulthood.

Some of the informants exhibited a profound understanding of how their childhood experiences had a lasting impact on their current health, particularly those who grew up in problematic family environments or experienced abuse and violence. They articulated an augmented vulnerability to new stressors in adulthood, manifesting in mental health challenges such as rumination, symptoms of anxiety and depression, posttraumatic stress disorder, and recurring contemplation of suicide. Additionally, many reported grappling with physical pain, fibromyalgia, sleep problems, exhaustion, and gastrointestinal problems.

One example is an informant mentioned earlier who grew up in a family with an alcoholic father and a mentally ill mother. She described experiences of mental health problems and panic attacks when she grew up, and an increased vulnerability to new stressful burdens in adulthood, such as getting pregnant:

“I have carried many of these things on my own then. So, when I had panic attacks, I didn't really know how to solve it, so I just compensated with different things and different behaviors. … then I went to some therapy sessions, and I got some antidepressants, and it went well for a couple of years. Then, when I got pregnant … I had a kind of psychosis. That's when all the stuff from childhood came back to me.” Group 2, nr. 1, female

She also connected this increased vulnerability to her reactions to new stressful events the year before her current sick leave, when she felt that “*everything fell apart*”*.* She experienced several crises in her family, including issues with her children and husband, and faced a stressful downsizing and relocation into a new job at the same time. She was diagnosed with depression and described her experiences as causing bodily pain, anxiety, and a feeling of emptiness. Acting upon her thoughts became an elusive task, and things seemed insurmountable.

Another informant, who endured childhood abuse and experienced extensive bullying from first through to ninth grade, has struggled with suicidal thoughts since she was 13 years old:

“I have a lot of baggage from school with bullying and everything, so it started already then. … Then you don't go to school anymore. …Our teacher sat in the classroom and the others came in and hit me, talked badly about me, and she didn't say a word. … it's still affecting me a lot.” Group 3, nr. 3, female

An additional example involves a female informant who, similarly, has a background marked by abuse and violence. She has received a diagnosis of posttraumatic stress disorder stemming from her exposure to numerous traumatic deaths spanning her life from childhood into adulthood. The challenges began when she was 3 years old, when she tragically lost her sister to crib death. Subsequently, she witnessed her uncle's suicide during a family gathering, and thereafter experienced a series of deaths in her close family for 10 consecutive years. At time of the interview, she grappled with the difficulties of her role at a nursing home, where she was confronted daily with the illness and demise of individuals under her care:

“When I tried to go to sleep, it was just flashing before my eyes. … you can't relax because there's so much chaos in your head. … I was supposed to go back to work with the old and the sick and the dying, … and I can't do it anymore. … I don't want to expose myself anymore to it and subject my body and mind to it. … anxiety and depression and post-traumatic stress, it settles in the neck and shoulders and the body aches.” Group 3, nr. 6

The importance of ACE for current health were also mentioned by some respondents when asked to describe and rank the three most important factor causing their health problems in an open-ended question (see Table 5).

**Table 5:** Distribution of negative life events in the context of own health, work, and private life across subgroups of participants based on their history of childhood and adult negative life events.

Negative life events	History of negative life events				*p*-value
	Group 1	Group 2	Group 3	Total	
	*N* (%)	*N* (%)	*N* (%)	*N* (%)	
Own disability					
Muscular issues and pains, including fibromyalgia	16 (47)	14 (54)	8 (40)	38 (48)	0.65
Mental distress	3 (9)	9 (36)	8 (40)	20 (25)	**0.01**
Fatigue, burnout, sleep	13 (38)	10 (39)	8 (40)	28 (35)	0.99
Other bodily ailments, rheumatic, genetic	11 (32)	10 (39)	4 (20)	25 (31)	0.40
Experience one or more of own disability problems	30 (88)	23 (89)	17 (85)	70 (88)	0.93
Negative life events in the context of work					
Too large workloads and demands	13 (38)	10 (39)	4 (20)	27 (34)	0.32
Excessive physical strain: physically demanding work, repetitive and/or heavy lifting	9 (27)	3 (12)	5 (25)	17 (21)	0.34
Relational problems: bullying, conflicts, collaboration issues	2 (6)	3 (12)	2 (10)	7 (9)	0.73
Concerns about losing the job due to health issues or reorganization	12 (35)	8 (31)	1 (5)	21 (26)	**0.04**
Resigned from job/lost job	2 (6)	4 (15)	3 (15)	9 (11)	0.43
Experience one or more negative life events in the context of work	24 (71)	19 (73)	12 (60)	55 (69)	0.61
Negative life events in the context of family and private relations					
Interpersonal challenges (mental abuse, conflicts, harassment, substance abuse)	2 (6)	7 (27)	7 (35)	16 (20)	**0.02**
Family pressures, including caregiving burdens and a disabled/ill child	6 (18)	5 (19)	3 (15)	14 (18)	0.93
Serious illness and death in close family	2 (6)	1 (4)	2 (10)	5 (6)	0.69
Divorce	2 (6)	1 (4)	3 (15)	6 (8)	0.33
Experience one or more negative life events in the context of family and private relations	8 (24)	9 (35)	7 (35)	24 (30)	0.55
Negative life events in the context of private life, other					
Accidents and complications following surgeries	9 (27)	3 (12)	2 (10)	14 (18)	0.19
Financial matters	1 (3)	2 (8)	2 (10)	5 (6)	0.55
Events as a child and unspecified life events	0 (0)	2 (8)	2 (10)	4 (5)	0.20
Experience one or more negative life events in the context of private life, other	10 (29)	5 (19)	4 (20)	19 (24)	0.59
Most important cause of health complaints reported by the participants					
Own disability the only one mentioned	7 (21)	8 (32)	6 (30)	21 (27)	0.57
Combination of work, private life and own disability	18 (53)	14 (56)	11 (55)	43 (54)	0.97
Work-related only	7 (21)	3 (12)	2 (10)	12 (15)	0.50
Don't know	2 (6)	0 (0)	1 (5)	3 (5)	0.48

Group 1: participants without adverse childhood experiences.

Group 2: participants with adverse childhood experiences, but not revictimized.

Group 3: participants with adverse childhood experiences and revictimized.

The difference between groups were evaluated by use of table analysis and the Pearson chi-square test. The differences across groups are statistically significant with *p*-values ranging from 0.05 to less than 0.001.

Significant differences are indicated in bold.

### Negative life events in the context of work affecting health

This theme relates to the participants’ experiences and perceptions on how exposures to different work factors negatively affected their health. The subthemes include work being too demanding due to either “a psychologically demanding work situation” or “a physically demanding work situation”. A psychologically demanding work situation is defined by the participants’ perceptions of the workload as too high, involvement in conflicts, feelings of incompetence, or uncertainty regarding their future job. A physically demanding work situation is defined by the participants’ perceptions of heavy, static or monotonous work. Additionally, experiences of “accidents at work causing health problems” are included.

### Perceptions of work as too demanding

Nearly all informants described physical and psychosocial workloads that exceeded their work capacity before sickness absence. About two-thirds of the respondents mentioned this when they had to rank three possible causes of their health problems (see Table 5). However, only a few rated work-related challenges as the sole cause of their complaints (15%).

Many informants described that they had continued to work in a physically demanding job despite experiencing health problems for many years until they could no longer manage. Several also felt that they had “hit the wall” due to high workload and demands in their job situations, or because of overcommitment and pushing for career aspirations. More than half of the informants (56%) also faced stress linked to job uncertainty. This stress arose from concerns about potentially losing their job due to health issues, workplace downsizing or restructuring, or because they had already been laid off or had decided to quit. There were no significant differences between the groups among the informants. However, we did find a difference in the analyses of the respondents’ answers when they had to prioritize reasons for their complaints (see Table 5). Here, 26% mentioned concerns about losing their job due to health issues or reorganizations, with participants in Group 1 having the most concerns (35%).

Interpersonal conflicts were identified as one of the most stressful experiences causing health problems among the participants. About 20% of the informants recounted facing such challenges, and 9% of the respondents reported this. The origins of these conflicts varied, encompassing disputes between employees and management, conflicts between management and union representatives, employee-to-employee conflicts, and even a legal dispute related to ownership in a private firm.

This is illustrated by one informant who held a management role, and who got into a conflict with an employee. She had been in a stressful work situation for several years because she was tasked with implementing numerous unpopular organizational changes without sufficient support from her superiors. Additionally, she had to address a challenging workplace culture, resulting in new employees feeling excluded from the group. This situation evolved into a complex case when she had to confront one of her employees at the core of this cultural challenge:

Regrettably, this individual, who eventually went on sick leave … organized an agenda against me as the leader, in terms of creating a letter … that was pure harassment. And it was sent, not to me, but to my superiors and the union representatives, … I felt so stabbed in the back. Especially given that it was labelled as concerning all employees. Then I went a bit downhill (voice breaks) and was already exhausted at that point. Group 1, nr. 1, female

### Negative life events in the context of private life affecting health

This theme relates to the participants’ experiences of private life being too demanding in adulthood, with the subthemes: “Experiences of severe violence and harassment”, “Family-related burdens” and “Worries and stress due to a strained economy.” Family-related burdens are defined as conflicts and breakups, substance abuse in close relations, caregiving due to sick children or parents, and deaths. Additionally, “Severe injuries from accidents or surgeries” causing ill health are included.

### Perceptions of private life as too demanding

#### Experiences of severe violence and harassment with a significant impact on health

Among the respondents, 20% mentioned interpersonal challenges as one of the main reasons for their current health problems, with a significantly higher occurrence in Group 3 (35%) and Group 2 (27%) compared to Group 1 (6%) (see Table 5). In total, 33% of the informants had experienced more severe forms of problems in close relationships during adulthood, such as physical and psychological violence and abuse, which affected their mental and physical health. This included all individuals in Group 3, 25% in Group 2 and 14% in Group 1. All of those who had experienced more severe forms of violence were separated from their spouses.

Examples of violence were ex-spouses who monitored and stalked the informants, obtained keys to their homes, and entered when they were away. Other informants recounted about spouses who had repeatedly ridiculed them, put them down and told them they were no good for many years before divorcing. A few had experienced severe physical violence.

An example is one of the revictimized informants who had been married to a manic-depressive and drug-dependent man. The physical violence in the marriage escalated to the point where her life and health were in danger. She suffered numerous physical injuries in the form of bruises and marks on her body, as well as more serious injuries that required hospital treatment. It eventually led to her fleeing one night with their shared child. After a few years, her spouse passed away. She felt that she could breathe easier, but her health problems intensified. She also faced financial difficulties, being responsible for her ex-husband's debt, alongside having a physically demanding job. All of this increased the burden and negatively impacted her health. At the time of the interview, she had extensive health issues:

“The physical bodily pains, particularly in the neck and a herniated disc in the back, have been quite significant, especially in the last five years. … (last year I got) a kidney infection and pneumonia,.. (they) found a blood clot in my right lung… I get very tired when I don't have a good night's sleep, and I’ve had these problems for three years. … I haven't really been motivated for anything. I've been in a kind of depression because everything hurts and is exhausting. And when I say that I am exhausted, I really mean it. After I've taken a shower, I'm so tired that I can sit on the bed for half an hour before I start getting dressed. And when I then get to work, I am completely (exhausted), I have nothing to give. That's how it has been in recent years at work.” Group 3, nr. 4, female

Many still experienced conflicts and harassment persisting long after divorce, especially when children were involved. In the most serious cases involving custody disputes, additional stress came from legal battles. Another of the revictimized informants said that her ex-spouse began harassing her about 10 years ago. Due to his behavior, she contacted child protection services, which led to a court case resulting in him having a restraining order for a period. After several rounds in court, he was granted regular visitation rights. Despite him having visitation rights, the harassment continued, and the overall burden became too much for her. Her ex-spouse framed it as a custody battle, but she felt that he was more focused on suppressing her than finding a solution for their child. She described the content of the harassment and her physical and mental reactions to it in this way:

“Many text messages pour in during a day. 5, 6, 7 of them. And long emails. He sends most of them at night… He has threatened to knock me down, and in that moment, I also get scared. And when I think about it afterwards, I think about the fact that he has such a low threshold for saying and doing such things to me, how is he really towards our son?

.. Regarding the neck and shoulders, the stiffness I experience, I believe it's connected to my mental state. I sleep poorly and can't relax properly. In terms of work, it's painful to deal with it at work. Additionally, I've been diagnosed with ‘wear and tear’ in my knees. And I have an old injury in my left leg.” Group 3, nr. 5, female

### A too demanding private situation due to family-related burdens

About a quarter of the informants (23%) and respondents (24%) reported other stressful family burdens, such as taking care of sick children and parents, deaths in close relations, substance abuse, less serious conflicts in close relations, or relationship breakdowns that had a psychological and physical impact at the time of the interviews. Even if it is not the most common problem, one in five informants have experienced substance abuse by their parents, spouses, or children. Some have experienced this in several of their close relationships. Among the most exposed, it has been a source of long-term stress and concern, also involving acute events related to violence, threats, and overdoses. As for this informant who had been in a marriage with an alcoholic and tried to cope for 25 years:

“I have probably been tense for many years because of this situation… There have been very many traumatic things that I have experienced. Towards the end now… he just collapsed on top of me and… Mixed pills and… It was just like… Is he even alive, really?… When I finally got away from him, I was just so relieved to get away from the situation… I was so exhausted… So, the first half a year, well… it went okay. … And then it hit me.” Group 2, nr. 4, female

### A strained economy an additional source of stress and worries

Half of the informants encountered financial constraints, with 25% expressing considerable economic concerns as source of stress. Notably, informants in Group 3 (89%) and Group 2 (50%) appeared to struggle more with financial issues compared to those in Group 1 (14%). Nearly all the informants who struggled were single. Among the respondents, only 6% mentioned their economic situation as one of the three most important reasons for their complaints, and we did not find any difference between groups.

The financial challenges reported by the informants stemmed from diverse factors, including high levels of indebtedness, diminished income resulting from small and/or unstable jobs relative to their financial obligations, or long-term illness and job loss leading to income reductions. Some had been in precarious employment or small positions for several years, often relying on extra shifts or additional positions to make ends meet, with women being disproportionately affected. When their health deteriorated, they found themselves unable to continue these practices of extra work, forcing them to make difficult decisions concerning their expenditures.

For most informants, the financial burden persisted over several years. A few had their finances regulated by the welfare system or knew that they should seek help to reduce the stress burden. The latter case is exemplified by a single mother who had accumulated significant debt over more than a decade due to a past break-up and ongoing daily expenses while caring for a child who had been very ill, periodically spending more time in the hospital than at home:

“… it was such that if I paid one bill, I had to let another one wait. So, during that time, I accumulated collection debt, … and it keeps growing. It's a stress factor like no other!… It's unmanageable for me. Plain and simple. I know I need to seek help for it, but I haven't mustered the energy for it yet.” Group 1, nr. 2

Her child didn't sleep at night because of high activity of his illness; thus, she didn't sleep either. However, because she needed the income, she persistently pushed herself to work for years, even when fatigued. The combination of her attempts to balance work, demanding caregiving, and managing a debt she couldn't handle took a toll on her health, resulting in issues such as hormone imbalances, rheumatism, fatigue, symptoms of depression, and prolonged and recurrent sickness absences.

Another illustrative case involves a male informant who has a history of childhood sexual abuse and physical violence. He identified the financial strain as a notable factor exacerbating his issues. The debt had accumulated several years ago due to a previous bankruptcy, yet his earnings continued to be garnished each month. He experienced a range of health ailments, including fibromyalgia, depression, and burnout. The additional burden of financial concerns made it even more challenging for him to manage the pain:

“I've ended up in a situation where things have gone really wrong for me financially, you know. … If you have some bright spots, something to look forward to in a way, it's easier to handle all of this, you know. But when everything seems dark, it's ten times harder to try to get out of this situation.” Group 2, nr. 5

### Severe injuries from accidents and complications related to surgery affecting health

One third of the informants had injuries from earlier accidents or complications after surgery that still affected their health. Additionally, 18% of the respondents mentioned it as one of the three most important reasons for their health problems. Typically, these were physical injuries from car accidents and other accidents related to leisure activities. Complications related to surgeries included nerve damage, infections, and hernias.

One example is an informant with a history of psychological and physical violence in childhood who experienced life-threatening complications when he underwent a stomach operation 5 years ago. These complications still have a serious impact on his health, especially causing sleeping problems, pain, and fatigue:

“Well, it all started with those stomach acid issues, which I began dealing with at a pretty young age. I had a terrible amount of stomach ulcers, and I also had a hiatal hernia. … First, they created a kind of loop to prevent all that pressure up in the esophagus. … (Just after) I went on holiday. … I got some huge pains in my stomach, and then all the stitches had come undone. … I had lost blood circulation in the top of my stomach. … (After arrival home) I had to undergo gastric bypass surgery.” Group 2, nr. 2

### Complex interactions between negative life experiences and health

This theme relates to the informants’ narratives and experiences of an accumulated health burden due to exposure to multiple negative life events throughout their lifespan. Two subthemes describing this complexity were identified: “The total burden is too high” and “The complexities of life events and the timespan involved make it difficult to understand the causes of their illnesses”.

### The total burden is too high

The total burden could be experienced as too high among the informants, either because they had been living with multiple problems for many years, or because many things occurred at the same time in a short timeframe. As already illustrated and supported by both the qualitative and quantitative material, there is seldom only one factor contributing to health issues among the participants. Among the respondents, more than half mentioned a combination of issues at work, in private life, and their own health (54%) (see Table 5). All the informants described several possible sources of ill health when going in depth on the reasons for their health problems. A common combination of issues includes conflicts in close relationships, financial struggles, persistent health problems, and stress arising from an inability to manage workplace demands, job loss, or feeling in danger of losing their job.

The intricacies of many individuals’ experiences are exemplified by the case of an informant who was sexually abused as child. He recently lost his job due to downsizing, leading to reduced income and financially struggles and worries. Simultaneously, he grappled with concerns about the continuity of his profession due to recurring back problems stemming from a work accident a few years ago, causing significant pain. Additionally, he encountered challenges in collaboration with his ex-wife, who had custody of their children. He perceived ongoing attempts to control and belittle him. Notably, he harbored anxieties about the well-being of their children, as his ex-wife oscillated in and out of psychiatric care and resided with a new partner who had displayed violence toward his son. At time of the interview, he was actively engaged in the process of seeking custody for their children, and the total burden was perceived as highly stressful:

“Most of all, I want to continue in the profession I have been doing for over twenty years. But it's not certain, it depends on my back… after I got the dismissal letter from work, my back only got worse and worse. …

I struggle financially. Not getting the money I'm used to… it's weighing on me a lot. …

The mother of my children goes in and out of psychiatric care, which is very stressful for me….

I am on my way to a court case in connection with the children… Because they are not doing well as things are today. All those things have an impact on what happens to me…” Group 3, nr. 2, male

### Complexities of negative life experiences and the timespan involved make it difficult to understand the causes of their illnesses

The difficulty for the participants to understand the causes of their illnesses can be illustrated by the respondents’ responses when they were asked to rank the three main causes of their health problems. More than a quarter of the respondents (27%) just reported their health complaints and no other reason. Some attributed their health problems to bad genes, mentioning other family members with the same illness. For others, it was just difficult to understand and pinpoint why they had their health problems.

One of the informants with a long history of NLEs in childhood and adulthood highlights why it is challenging for many to specify a particular cause for their health issues when answering the question, “Do you have any idea what a triggering cause for your issues could be?”:

“No, I don't know… It just manifested in my body, so I don't know what… But I have worked a lot over the years, you know. Cleaning …, so I think that's what caused the muscle problems and such. When it comes to the mental aspect, well, it's… everything I've been through with my son (a drug addict) … It's kind of from childhood, and you go into depressions and get those negative thoughts about things you've experienced.. I've had a tough life myself also you know… there are many factors at play.” Group 3, nr. 7, female

## Discussion

The aim of this study was to increase our understanding of the complexities faced by patients referred to occupational rehabilitation, specifically regarding ACEs and NLEs throughout their lives, and their impact on health.

We found that about half of the participants had experienced ACEs. Common ACEs reported by the participants were neglect, psychological violence, physical violence, and sexual abuse. In the subset of participants who had been subjected to any form of violence or abuse in childhood, nearly two-thirds reported experiencing further victimization during adolescence or adulthood. Moreover, these individuals had experienced significantly more negative events throughout life compared to those without ACEs, or those with ACEs but not revictimized. Revictimization were more prevalent among participants from dysfunctional families. Women are often overrepresented when it comes to sexual abuse (2). However, in our data, there is an equal distribution between genders.

All participants had a high symptom burden, but those with a history of ACEs reported more mental health issues than those without ACEs. However, there were no significant differences in mental health issues between the two ACE groups. In adulthood, NLEs both in the context of work and private life were affecting health. Most mentioned a combination of factors. Nearly all informants described physical or psychosocial factors in work that exceeded their work capacity. Stressors related to private life that influenced their health, were especially psychological violence, intimate partner violence, conflicts in relationships and financial struggles. These problems were notably more common among those exposed to ACEs compared to non-exposed individuals, and especially among revictimized. While symptoms of anxiety and depression were more prevalent among those with ACEs, attributing ACEs as the sole cause remains uncertain. Analysis of the informants’ life histories and their perceptions of the causes of their health problems revealed complex issues, illuminating the interactions between NLEs, health issues and the unique context of each individual, often involving personal issues, work and private life. Some informants attributed ACEs directly to their health issues, while others, experiencing numerous NLEs in both childhood and adulthood, found it challenging to pinpoint a specific cause. They only acknowledge the manifestation of these experiences in their bodies. However, several of the informants with ACEs noted increased vulnerability to new stressors as adults, aligning with the stress sensitization hypothesis (15, 55). Repetition of stressors from childhood into adulthood can activate mechanisms that affect coping resources and coping strategies and stimulate appraisal of subsequent stressors as uncontrollable (56). Prior adversity exposures are also associated with altered adult brain reactivity to diverse challenges, which might diminish the persons’ ability to cope with later stressors and produce enduring susceptibility to mental health problems (57).

### Comparison with other studies

To the best of our knowledge, our study is the first to report the prevalence of ACEs and NLEs among participants attending occupational programs. Therefore, a direct comparison of prevalence in the same population is not possible. Nevertheless, when juxtaposed with the WHO's global child maltreatment report, our study indicates somewhat lower rates of psychological violence as mentioned in the compared to the WHO regions of Americas (38% vs. 24%) (2), but somewhat higher rates than the results reported in the WHO regions of Europe and in a Norwegian population study (15%) (58). The prevalence of neglect among the participants in our study is threefold the results for the WHO regions of Europe and in the Norwegian population study (58). Meanwhile, rates of physical violence and sexual abuse in our study closely align with both the WHO report and the findings in the adult Norwegian population. Our observation that maltreatment is more common in families with parental drug or mental health issues is consistent with other studies (59, 60).

Individuals with ACEs in our study showed an increased vulnerability to new events in adulthood. This vulnerability was particularly evident in experiences of revictimization, interpersonal challenges, and financial difficulties, also aligning with findings in other studies (9, 10, 12, 61, 62). While the overall exposure to violence or abuse in adulthood is similar between our participants and findings in the adult Norwegian population (36% vs. 40%), the proportion experiencing both childhood violence and new victimization in adolescence/adulthood is three times higher in our study (62% vs. 20%) (58). However, the proportion of participants grappling with significant financial challenges is comparable to the adult Norwegian population (25% vs. 20%) (63).

Regarding identified stressors in adult life impacting health in our study—specifically psychological violence, intimate partner violence, conflicts in relationships, and financial problems—harmonizes with the findings of the Norwegian quality of life surveys in 2020 and 2021 (62). These studies linked financial problems and psychological violence to the highest short-term mental health risks, with all forms of violence increasing the risk of reduced mental health. While financial issues often result from other negative life events, such as divorce, job loss, or prolonged sickness absence, studies identify financial issues as independently associated with severe health effects (30, 64). Most of the participants in our study are also working in occupations which are associated with high sickness absence due to musculoskeletal disorders, such as manual occupations and service and care workers (65).

## Strengths and limitations of the study

The model used in our study has several advantages. We made a classification into three subgroups to capture more of the total stress load among those exposed to violence in both childhood and adulthood when investigating differences in the total number of ACEs and NLEs and differences in health. This contrasts with other research where the common approach uses a cumulative risk model, either employing continuous variables or constructing categorical variables to measure the impact of polyvictimization on different outcomes (4, 19). Increasingly, latent class analysis is used to explore how different combinations of ACEs affect children, adolescents, and adults differently, for example, considering health outcomes (66). This approach accounts for the complexities of exposures and potential overlap between various types of maltreatment and household dysfunction. However, none of these approaches take revictimization into the classifications. Studies also vary in defining and measuring adversity components and types (67). For example, to enhance the original ACEs-scale, suggestions include incorporating important predictors of physical and mental health problems, such as peer victimization, peer rejection and exposure to community violence (68). The number of items included in the model, and how they are measured will impact the results.

Our model allows for ACEs exposures to overlap, implying that the non-revictimized group may share many childhood stressors with the revictimized group. The chosen classification effectively differentiated those most exposed to stress in both childhood and adulthood, but there were generally minor differences in health outcomes between the two groups. With a larger sample, additional subgroup divisions, including more negative exposures both in childhood and adulthood, might better explain variance in occupational rehabilitation-relevant health outcomes. Although a limitation of our study is the small number of participants, the analytical model still revealed differences between the three groups, particularly in terms of exposure to negative life events that can affect health, as well as differences in mental health.

A strength of our study is the mixed-methods approach, allowing more in-depth knowledge and contextual understanding of the complex issues of patients referred to occupational rehabilitation. We have a high response rate across survey questions, coupled with interviews conducted with fifty percent of the respondents. This dual approach affords a more comprehensive perspective on ACEs and NLEs, elucidating their implications on health. In addition, since the results from different methods converge and support each other, it strengthens the results.

There are also several limitations to this study. People who volunteer to be interviewed often differ from others in important ways, e.g., by expressing more troubled experiences than those who do not (39). However, we do not find significant differences between individuals who were interviewed and those non-interviewed. Even though, we cannot exclude that those who agreed to participate in the study have more troubled experiences than those who declined. Some of the analyses are based on a small number of individuals, which may contribute to the possibility that some significant differences could be due to chance.

Regarding the questionnaire used, there is a potential recall bias in self-reported ACEs information, but false positives are probably rare (69). Although some underreporting was identified, the convergence of data methods enhances the overall credibility of the results. We have used a shortened and modified version of the ACEs questionnaire due to ethical constraints, limiting specificity. More detailed questions on adversity severity and duration and including more childhood burdens both inside and outside family, could have enhanced the study. The questions used to measure NLEs in adulthood are also customized for this study. A weakness in the NLEs questions, is that we cannot distinguish between different forms of violence and abuse after the age of 18 or differentiate between bullying before and after 18 years of age. The use of standardized and validated questionnaires could have contributed to more comparable results across studies. Simultaneously, our qualitative approach provided contextual information regarding both ACEs and NLEs, along with their significance on participants’ health. This, to some extent, mitigates the limitations of the questionnaire.

The study did not investigate resilience factors that may reduce the impact of ACEs on adult health, such as social support, coping and socioeconomic resources, and stressor solvability and coping, which could enhance the understanding of health variations (56, 70, 71). Benevolent childhood experiences may counteract the negative effect of ACEs in psychological problems (72). Prior life events in childhood can also lead to resilience building and buffer the detrimental effects of stress (71). Inclusion of resilience factors may have given greater understanding of variations in results across groups.

### Implication for research and rehabilitation

The findings of our study underscore the potential impact of negative life events on health. In order to identify effective interventions, it is crucial to monitor the prevalence of such events and evaluate if and how they are affecting the patients’ health and ability to work (4). Norwegian clinicians working in occupational rehabilitation, typically work in interdisciplinary teams and conduct a comprehensive biopsychosocial screening at the initiation of the intervention (73). This screening aims to elucidate individual complexities, allowing for tailored RTW-interventions. However, to our knowledge, even if the clinicians regularly encounter patients with ACEs and other NLEs, they do not screen for it in a systematic way, and it is seldom addressed as a part of their interventions. It is also a notable challenge that ACEs are seldom addressed in standard follow-up consultations within the specialist health care system and among general practitioners (74). Many individuals often experience a prolonged cycle of ineffective consultations and treatments. In some cases, the treatment they receive can even contribute to deterioration, suggesting that the root causes of the problems may not be addressed adequately.

Many clinicians refrain from inquiring about ACEs and other private life events that may act as barriers to return to work for several reasons. One reason is the potential burden, especially for the patient, associated with addressing such topics. Another challenge lies in how to handle the knowledge gained about the patient, especially given that the interventions provided are not designed to delve deeply into various individual issues related to privacy and health. Lack of knowledge about the topics, both regarding how they affect health and what can be done to assist participants, can be additional barriers. However, a study investigating the acceptability of a screening for potentially traumatic life events among patients in a Norwegian outpatient pain clinic, found that most patients are not only willing to complete such assessments but welcome them (75). Also, most of the participants had a desire to be met with a holistic treatment approach and indicated that the screening enabled them to effectively explore the interaction between their pain condition and the traumatic event(s) within a clinical setting. Common concerns included doubts about the clinics’ ability to follow-up on the disclosed information. Some experienced a temporary increase in trauma-related thoughts, but none reported an inability to manage them. The researchers concluded that a brief trauma screening could be helpful to guide clinical practice in chronic pain settings. They also suggested that the high level of comorbidity and interrelated causal mechanisms of chronic pain and PTSD indicate that an integrated treatment approach in pain clinics may be preferable.

Stressful events, whether they occur in the family, at school, at work, or in other places, may affect adult health and work ability, but they can be treated. Sapolsky suggests that even if early life adversities can leave broad and permeating scars of neurobiological dysfunction, there is considerable potential to mitigate the negative consequences of ACEs in adults (76). Findings also indicate that interpersonal relationships, cognitive vulnerabilities and behavioral difﬁculties in adulthood may be modiﬁable predictors of depression following maltreatment (77). However, interpersonal issues are pointed at as a possible overlooked factor negatively influencing RTW among patients in occupational rehabilitation (78). Financial problems may represent another overlooked factor affecting many, and that requires screening and proper follow up. For persons with multiple problems restricting their work ability, a focus on health and medical barriers to work is too narrow, because personal, social, and environmental factors might also obstruct participation in work (30). There is a need for a holistic and individualized approach (79).

## Conclusion

A substantial proportion of those referred to occupational rehabilitation have experienced ACEs. Compared to those without ACEs, it appears that they are more susceptible to facing new events in adulthood, such as problems in interpersonal relations and financial issues, especially revictimized individuals. Additionally, both the ACEs groups are struggling more with their mental health than those without ACE experiences. It is strongly recommended to screen for ACEs and other NLEs among patients referred to occupational rehabilitation, recognizing the potential to reduce, stop or even reverse the adverse consequences of ACEs in adults. This might be important to help this patient group return to work. Further research is recommended to investigate the prevalence, types, and combinations of different life events in childhood, adolescence and adulthood affecting health and work participation among those receiving sickness absence and work disability benefits. We recommend a holistic approach to develop effective interventions according to individual needs.

## Data Availability

The data can be made available upon approval from the Regional Committees for Medical and Health Research Ethics in Norway (Grant number: 2013126). Requests to access the datasets should be directed to the corresponding author.
